# A real-world pharmacovigilance study of FDA Adverse Event Reporting System (FAERS) events for venetoclax

**DOI:** 10.1371/journal.pone.0278725

**Published:** 2022-12-07

**Authors:** Yang Yang, Yamin Shu, Guosong Chen, Yanchao Yin, Feie Li, Juan Li

**Affiliations:** Department of Pharmacy, Tongji Hospital, Tongji Medical College, Huazhong University of Science and Technology, Wuhan, 430030, China; Auburn University, UNITED STATES

## Abstract

**Background:**

Venetoclax (VEN) is the first selective small molecule Bcl-2 inhibitor approved by FDA and used in adult chronic lymphocytic leukemia (CLL), small lymphocytic lymphoma (SLL) and some acute myeloid leukemia (AML). However, the long-term safety of VEN in large sample population was unknown. This study evaluated the adverse events (AEs) of VEN from FDA Adverse Event Reporting System (FAERS) since its approval in 2016 by data mining.

**Methods:**

The disproportionality analyses, including four algorithms of reporting odd ratio (ROR), proportional reporting ratio (PRR), bayesian configuration promotion neural network (BCPNN), and multi item gamma poisson shrinker (MGPS), were employed to quantify the signals of VEN-associated AEs.

**Results:**

From the FAERS database, a total of 8,379,682 reports were collected during the study period. After removing the duplication, the number of reports with VEN as the primary suspect (PS) was 19,107. The 19,107 cases of AEs involved 27 organ systems, 256 significant PTs which conforming to the four algorithms. Unexpected serious AEs, such as pleural effusion, splenic infarction, atrial fibrillation, skin squamous cell carcinoma, etc., have signals. The median time of occurrence of AEs related to VEN was 31 days (inter quartile range [IQR] 7–131 days), and half of the reported AEs occurred within 1 month after administration.

**Conclusion:**

Our research has found new significant AEs signals of VEN, which improved its safety information in real-world after marketing approval, and contributed to its risk control of use in clinic.

## 1. Introduction

VEN is the world’s first oral, small molecule, selective inhibitor of B-cell lymphoma factor-2 (Bcl-2) for clinical use, which has been approved by FDA in 2016 for the treatment of adult chronic lymphocytic leukemia (CLL) or small lymphocytic lymphoma (SLL), and then approved in many countries and regions including the European Union, Canada and China [1,2]. In 2018, VEN was approved by FDA to combine azacytidine, or decitabine, or low-dose cytarabine for the treatment of newly diagnosed acute myeloid leukemia (AML) in adults 75 years or older, or who have comorbidities that preclude use of intensive induction chemotherapy [2].

Bcl-2 is one of the most important anti-apoptotic proteins in hematological malignancies, which can inhibit the recruitment of BAX and BAK, and block the activation of apoptotic pathway by binding pro-apoptotic proteins [1]. Abnormal expression of Bcl-2 family proteins is often found in hematological malignancies, the most important of which is the over expression of Bcl-2. High levels of Bcl-2 expression were found in the patients of follicular lymphoma (FL), chronic lymphocytic leukaemia (CLL), mantle cell lymphoma (MCL) and Waldenstrm’s macroglobulinaemia (WM) [3,4]. Therefore, Bcl-2 inhibitors can be used to block binding sites, release pro-apoptotic proteins, restore blocked apoptotic pathways and promote apoptosis [5,6]. Although the early non-selective Bcl-2 inhibitor has shown good antitumor activity in clinical trials, it can also act on Bcl-X_L_, which will lead to severe thrombocytopenia [7,8]. As a small molecule targeted drug, selective Bcl-2 inhibitors VEN overcome the problem of thrombocytopenia [9,10], which brings new treatment options to patients with hematological malignancies.

The short-term clinical trials have reported significant AEs, including tumor lysis syndrome (TLS), neutropenia, thrombocytopenia and infections, and other common AEs include anemia, diarrhea, nausea, upper respiratory tract infection, cough, musculoskeletal pain, etc [11,12]. However, the sample size is relatively small, and the follow-up duration and observable AEs are limited. The long-term use of VEN may present previously unrecognized or serious safety concerns. Moreover, the time to onset of AEs was unknown in previous studies. Therefore, it is of great significance and necessity to explore the potential AEs of VEN through data mining algorithm by large-sample post-marketing monitoring.

FDA adverse event reporting system (FAERS) is a public database of spontaneously reported adverse drug events, which is updated every three months [13]. We mine the data obtained from the FAERS database to evaluate the AEs risks of VEN use in clinic, which provide more information for the safety of VEN after approval, and guide its clinical use more safely.

## 2. Methods

### 2.1 Data sources

The data of this study are from the FAERS database. Since VEN was approved in April 11, 2016, we derived the data from the second quarter of 2016 (2016 Q2) to 2021 from FAERS. The FAERS data file contains demographic and administrative information (DEMO), drug information (DRUG), adverse drug reaction information (REAC), patient outcome information (OUTC), information on report sources (RPSR), drug therapy starts dates and end dates (THER), and indications for use/diagnosis (INDI). Since the database is submitted by various sources, it may sporadically include duplicate reports, so it needs to be reprocessed. The CASEID and PRIMARYID were chosen as the key filter in our study to remove duplicate records, by selecting the latest FDA_DT when CASEID were the same, and choosing the higher PRIMARYID when the CASEID and FDA_DT were the same [14], resulting in a reduction in the number of reports to 8,379,682.

### 2.2 Adverse events and drug identification

The screened AEs are standardized and classified through the system organ class (SOC) and preferred term (PT) of the international medical dictionary for regular activities (MedDRA 24.0), and SOC and PT are taken as the objects of data analysis and research. After importing the original data of FAERS into MySQL software, we used both generic name and brand name including "venetoclax", "venclexta" and "venclyxto" as the drug name keywords. Moreover, in order to improve accuracy, we choose the role_cod as“PS”(primary suspected) in the DRUG files.

### 2.3 Data mining

The disproportionality analysis is an important analytical tool in pharmacovigilance research, which is used to compare the proportion of the target drug-related and other drugs-related AEs in order to study the correlation between the target drug and the target AEs. In our study, the reporting odds ratio (ROR), the proportional reporting ratio (PRR), the Bayesian confidence propagation neural network (BCPNN) and the multi-item gamma Poisson shrinker (MGPS) algorithms were used to quantify the signals of VEN-related AEs. The equations and criteria for the four algorithms are as described and detailed in **S1 Table**. A signal was considered when the lower limit of the 95% CI of the ROR exceeded one, or when the PRR greater than or equal to two and χ2 greater than or equal to four, or when the lower limit of 95% CI of the IC exceeded zero, or the lower limit of 95% CI of EBGM exceeded two, with the number of reports greater than or equal to three [15]. In general, when the specific AE occurrence rate of a specific drug is significantly higher than the background frequency in the database and reaches a certain threshold or criteria, and the higher the value of the four parameters, the stronger the signal. In this study, we selected AEs signals that satisfy the above four algorithm criteria at the same time in PT level, and signals can be detected when at least one of the four indicators meets the criteria in SOC level. Unexpected signals were defined as significant AEs found not listed in any labels.

### 2.4 Statistical analysis

Descriptive analysis and time to onset analysis were performed to study the characteristics of reports. The calculation method of time to onset of AEs is as follows: Time to onset = AEs onset date—VEN administration date. Typing errors or non-detailed reports, such as AEs earlier than the start date, need to be excluded. The duration of onset was described with medians and inter quartile range. All data processing and statistical analysis were performed by MYSQL 8.0, Navicat Premium 15, Microsoft EXCEL 2019 and GraphPad Prism 8.

## 3. Results

### 3.1 Descriptive characteristics

This study obtained the data from 2016 Q2 to 2021, and a total of 8,379,682 reports were submitted to FAERS. After deduplication and screening, the number of reports with VEN as PS was 19,107. In this study, the PS report data has been analyzed, and the detailed characteristics of the related AEs are shown in **Table 1**. Among the reported AEs, males (59.96%) accounted for far more than females (34.34%). In terms of age distribution, although the proportion of unknown age reached 43.25%, the AEs of the elderly (that is, over 65 years old) still accounted for a higher proportion (38.66%), reaching 7386. Most of the reported indications were leukaemia (71.94%), followed by lymphoma (8.15%), plasma cell myeloma (3.77%), myelodysplastic syndrome (3.70%) and globulin related diseases (0.36%). Most of the report sources are the United States (63.44%), and the others are less than 5%, such as France (3.56%), Canada (2.84%), Britain (2.36%) and Germany (2.20%). Interestingly, the proportion of non-health professionals (45.89%) is slightly lower than that of health professionals (53.97%) among the reporters. The highest proportion of consumers among non-healthcare professionals reached 45.98%, which exceeded that of physicians (40.35%) among health professionals, while only 3.46% of pharmacists. The annual report volume has been increasing year by year and tending to be stable, accounting for 2.16%, 8.04%, 15.99%, 21.98%, 24.42%, and 27.41% from 2016 to 2021. Compared with other serious medical events, accounting for 33.98%, the main proportion reached 75.67%, most of which were hospitality (39.02%) and death (33.92%). This may be related to the disease progression of this type of tumor. Notably, there were 8 cases of congenital anomaly, although the incidence was relatively low below 0.1%.

**Table 1:** Characteristics of reports associated with venetoclax from 2016 Q2 to 2021.

Characteristics	Case number(n)	Case proportion(%)
**Number of events**	19,107	
**Gender**		
Female	6,561	34.34
Male	11,456	59.96
Unknown	1,090	5.70
**Age**		
<18	135	0.71
18≤ and≤65	3,322	17.39
>65	7,386	38.66
Unknown	8,264	43.25
**Indications (TOP five)**		
Leukaemia	13,746	71.94
Lymphoma	1,557	8.15
Plasma cell myeloma	720	3.77
Myelodysplastic syndrome	707	3.70
Globulin related diseases	69	0.36
**Serious Outcome**		
Death	6,481	33.92
Life-threatening	444	2.32
Hospitalization	7,456	39.02
Disability	78	0.41
Congenital Anomaly	8	< 0.10
Required intervention to prevent permanent impairment/damage	1	< 0.10
Other serious medical events	6,492	33.98
**Reported Countries (Top five)**		
America	12,121	63.44
France	680	3.56
Canada	542	2.84
Britain	451	2.36
Germany	420	2.20
Reported Person		
health profession		
Physician	7,709	40.35
Pharmacist	662	3.46
health-professional	996	5.21
other health-professional	945	4.95
non-healthcare professional		
Consumer	8786	45.98
Lawyer	2	<0.10
Unknown	7	<0.10
Reporting year		
2021	5,237	27.41
2020	4,665	24.42
2019	4,200	21.98
2018	3,055	15.99
2017	1,537	8.04
2016 Q2 ^a^	413	2.16

^a^ The second quarter of 2021.

### 3.2 Signal values

Signal values of reports associated with VEN in SOC level are shown in **Table 2**. Statistically, we found that VEN-related AEs were distributed in 27 organ systems. Among them, there were 8 significant SOCs that met at least one of the four calculation criteria, including general disorders and administration site conditions (SOC: 10018065, 8,962), infections and infestations (SOC: 10021881, 4,458), blood and lymphatic system disorders (SOC: 10005329, 4,436), investigations (SOC: 10022891, 3,696), respiratory, thoracic and mediastinal disorders (SOC: 10038738, 3,378), neoplasms benign, malignant and unspecified (incl cysts and polyps) (SOC: 10029104, 3,142), surgical and medical procedures (SOC: 10042613, 2,299), metabolism and nutrition disorders (SOC:10027433, 1,680).

**Table 2:** Signal values of reports associated with venetoclax at the SOC level.

SOC	Case number (n)	ROR (95% two-sided CI)	PRR (χ^2^)	IC (CI025)	EBGM (EBGM05)
General disorders and administration site conditions	8,962	1.37 (1.34–1.41)*	1.20 (482.97)	0.26 (0.23)*	1.20 (1.16)
Infections and infestations	4,458	2.43 (2.35–2.51)*	2.09 (2857.40)*	1.06 (1.01)*	2.09 (2.02)*
Blood and lymphatic system disorders	4,436	5.91 (5.72–6.12)*	4.77 (13765.88)*	2.24 (2.19)*	4.73 (4.58)*
Injury, poisoning and procedural complications	4,242	0.69 (0.67–0.72)	0.76 (447.63)	-0.39 (-0.44)	0.76 (0.74)
Investigations	3,696	1.85 (1.79–1.92)*	1.69 (1164.41)	0.75 (0.70)*	1.68 (1.63)
Respiratory, thoracic and mediastinal disorders	3,378	1.28 (1.23–1.33)*	1.23 (166.84)	0.30 (0.24)*	1.23 (1.18)
Neoplasms benign, malignant and unspecified (incl cysts and polyps)	3,142	2.33 (2.24–2.42)*	2.11 (1983.87)*	1.07 (1.02)*	2.11 (2.03)*
Gastrointestinal disorders	2,756	0.76 (0.73–0.79)	0.79 (184.35)	-0.34 (-0.39)	0.79 (0.76)
Surgical and medical procedures	2,299	3.96 (3.79–4.14)*	3.61 (4443.96)*	1.84 (1.78)*	3.58 (3.43)*
Vascular disorders	2,146	0.70 (0.67–0.73)	0.73 (252.87)	-0.45 (-0.52)	0.73 (0.70)
Nervous system disorders	2,085	0.46 (0.44–0.48)	0.52 (1178.96)	-0.95 (-1.01)	0.52 (0.50)
Cardiac disorders	1,935	0.89 (0.85–0.93)	0.90 (25.56)	-0.16 (-0.23)	0.90 (0.86)
Metabolism and nutrition disorders	1,680	1.25 (1.19–1.32)*	1.23 (78.59)	0.30 (0.23)*	1.23 (1.17)
Musculoskeletal and connective tissue disorders	1,396	0.56 (0.53–0.60)	0.60 (434.13)	-0.75 (-0.82)	0.60 (0.57)
Skin and subcutaneous tissue disorders	1,365	0.40 (0.38–0.42)	0.44 (1152.55)	-1.18 (-1.26)	0.44 (0.42)
Renal and urinary disorders	1,021	0.77 (0.72–0.82)	0.78 (66.03)	-0.35 (-0.45)	0.78 (0.74)
Psychiatric disorders	986	0.37 (0.34–0.39)	0.40 (1023.16)	-1.32 (-1.42)	0.40 (0.37)
Immune system disorders	655	0.33 (0.31–0.36)	0.36 (839.52)	-1.49 (-1.60)	0.36 (0.33)
Hepatobiliary disorders	413	0.81 (0.74–0.89)	0.82 (17.65)	-0.30 (-0.44)	0.82 (0.74)
Eye disorders	284	0.34 (0.30–0.38)	0.35 (362.26)	-1.52 (-1.70)	0.35 (0.31)
Reproductive system and breast disorders	204	0.24 (0.21–0.27)	0.25 (493.17)	-2.02 (-2.23)	0.25 (0.21)
Ear and labyrinth disorders	162	0.66 (0.56–0.77)	0.66 (28.89)	-0.61 (-0.83)	0.66 (0.57)
Endocrine disorders	162	0.32 (0.28–0.38)	0.33 (229.87)	-1.61 (-1.84)	0.33 (0.28)
Social circumstances	100	0.44 (0.36–0.54)	0.45 (69.88)	-1.17 (-1.46)	0.45 (0.37)
Congenital, familial and genetic disorders	52	0.47 (0.36–0.61)	0.47 (31.42)	-1.10 (-1.50)	0.47 (0.36)
Product issues	40	0.05 (0.03–0.06)	0.05 (771.07)	-4.35 (-4.81)	0.05 (0.04)
Pregnancy, puerperium and perinatal conditions	24	0.07 (0.05–0.10)	0.07 (307.24)	-3.86 (-4.45)	0.07 (0.05)

*Indicates statistically significant signals in algorithm. SOC, system organ class; ROR, reporting odds ratio; CI, confidence interval; PRR, proportional reporting ratio; χ^2^, chi-information component; IC, information component; IC025, the lower limit of 95% CI of the IC; EBGM, empirical Bayesian geometric mean; EBGM05, the lower limit of 95% CI of EBGM.

By calculating the signal values of reports associated with VEN at the level of PTs, 256 PTs with significant disproportionality were found to meet the four calculation criteria at the same time, which were shown in **S2 Table**. After manual inspection, 75 major PTs were obtained after excluding those apparently unrelated to drug use and the number of reports was less than 30. See **Table 3** for details. The SOCs included in AEs of the drug label include blood and lymphatic system disorders, infections and infestations, investments and metabolism and nutrition disorders. PTs that have appeared in clinically reported AEs include neutropenia (PT:10029354), febrile neutropenia (PT:10016288), thrombocytopenia (PT:10043554), pyrexia (PT: 10037660), pneumonia (PT:10035664), infection (PT:10021789), sepsis (PT: 10040047), platelet count decreased (PT:10035528), white blood cell count decreased (PT:10047942), tumour lysis syndrome (PT:10045170). It is worth noting that unexpected important AEs included splenomegaly (PT:10041660), atrial fibrillation (PT: 10003658), splenic infarction (PT: 10041648), spleen atrophy (PT: 10076915).

**Table 3:** Signal strength of AEs reports associated with venetoclax at the PT level.

SOC	PT	Case number (n)	ROR (95% two-sided CI)	PRR (χ^2^)	IC (IC025)	EBGM (EBGM05)
Blood and lymphatic system disorders	Neutropenia	768	7.05 (6.56–7.59)	6.81 (3771.94)	2.74 (2.63)	6.72 (6.25)
	Febrile neutropenia	553	10.31 (9.47–11.23)	10.04 (4414.75)	3.27 (3.15)	9.84 (9.03)
	Thrombocytopenia	373	4.07 (3.67–4.51)	4.01 (839.08)	1.98 (1.83)	3.98 (3.59)
	Pancytopenia	341	7.89 (7.08–8.79)	7.76 (1979.06)	2.90 (2.74)	7.65 (6.86)
	Lymphadenopathy	262	9.24 (8.17–10.45)	9.13 (1860.59)	3.12 (2.93)	8.96 (7.92)
	Cytopenia	150	14.57 (12.38–17.16)	14.47 (1821.20)	3.68 (3.44)	14.04 (11.92)
	Splenomegaly*	94	9.07 (7.39–11.13)	9.03 (658.19)	3.02 (2.72)	8.87 (7.23)
	Bone marrow failure	88	3.69 (2.99–4.55)	3.68 (170.27)	1.81 (1.50)	3.65 (2.96)
	Myelosuppression	79	6.65 (5.32–8.31)	6.63 (372.17)	2.60 (2.27)	6.54 (5.24)
	Autoimmune haemolytic anaemia	65	18.31 (14.28–23.48)	18.26 (1017.90)	3.79 (3.42)	17.56 (13.70)
	Platelet disorder	43	19.26 (14.19–26.15)	19.22 (711.49)	3.69 (3.24)	18.45 (13.59)
	Haemolytic anaemia	38	5.31 (3.86–7.32)	5.30 (131.17)	2.21 (1.73)	5.25 (3.81)
	Bone marrow disorder	36	16.10 (11.54–22.46)	16.07 (490.93)	3.44 (2.95)	15.54 (11.14)
	Haemolysis*	34	5.02 (3.58–7.04)	5.01 (108.01)	2.12 (1.62)	4.97 (3.54)
	Blood disorder	31	4.72 (3.31–6.72)	4.71 (89.66)	2.02 (1.50)	4.67 (3.28)
Cardiac disorders	Atrial fibrillation*	238	2.84 (2.50–3.23)	2.82 (279.12)	1.47 (1.28)	2.81 (2.47)
General disorders and administration site conditions	Death*	4377	6.84 (6.61–7.08)	5.50 (16632.74)	2.44 (2.40)	5.45 (5.27)
	Pyrexia	846	2.99 (2.79–3.20)	2.90 (1062.76)	1.53 (1.42)	2.89 (2.69)
Immune system disorders	Immunodeficiency*	82	5.79 (4.66–7.21)	5.77 (319.49)	2.42 (2.09)	5.71 (4.59)
Infections and infestations	Pneumonia	957	3.36 (3.15–3.58)	3.24 (1494.27)	1.68 (1.59)	3.22 (3.02)
	Infection	529	4.21 (3.86–4.60)	4.12 (1248.74)	2.02 (1.90)	4.10 (3.75)
	Sepsis	488	5.05 (4.61–5.53)	4.94 (1526.40)	2.28 (2.15)	4.90 (4.48)
	Septic shock	142	3.84 (3.26–4.54)	3.82 (293.80)	1.89 (1.64)	3.80 (3.22)
	Fungal infection	81	2.69 (2.16–3.35)	2.68 (85.26)	1.37 (1.05)	2.67 (2.15)
	Diverticulitis*	71	2.72 (2.16–3.44)	2.72 (76.70)	1.38 (1.04)	2.71 (2.14)
	Clostridium difficile infection	70	2.97 (2.35–3.76)	2.97 (90.79)	1.50 (1.16)	2.95 (2.33)
	Pneumonia fungal	64	22.86 (17.77–29.40)	22.79 (1267.33)	4.02 (3.65)	21.71 (16.88)
	Localised infection*	63	2.73 (2.13–3.50)	2.73 (68.48)	1.38 (1.01)	2.71 (2.12)
	Bacteraemia	61	6.14 (4.77–7.91)	6.13 (258.17)	2.46 (2.09)	6.06 (4.70)
	Bacterial infection	50	3.38 (2.55–4.46)	3.37 (82.74)	1.65 (1.24)	3.35 (2.54)
	Skin infection*	43	4.17 (3.08–5.63)	4.16 (102.22)	1.91 (1.47)	4.13 (3.06)
	Neutropenic sepsis	42	6.85 (5.05–9.30)	6.84 (206.24)	2.54 (2.09)	6.75 (4.97)
	Pneumocystis jirovecii pneumonia	35	3.28 (2.35–4.57)	3.27 (54.81)	1.57 (1.08)	3.25 (2.33)
	Bronchopulmonary aspergillosis	34	4.99 (3.55–6.99)	4.98 (106.94)	2.11 (1.61)	4.93 (3.52)
	Aspergillus infection	33	4.71 (3.34–6.63)	4.70 (95.15)	2.03 (1.52)	4.66 (3.31)
	Escherichia infection	33	4.79 (3.40–6.75)	4.78 (97.66)	2.05 (1.55)	4.74 (3.36)
	Vascular device infection*	32	8.43 (5.94–11.97)	8.42 (205.33)	2.72 (2.20)	8.28 (5.83)
	Bacterial sepsis	30	11.41 (7.94–16.40)	11.39 (277.30)	3.02 (2.49)	11.13 (7.74)
Injury, poisoning and procedural complications	Off label use*	2339	3.03 (2.91–3.17)	2.79 (2782.97)	1.47 (1.41)	2.77 (2.66)
Investigations	Platelet count decreased	920	10.70 (10.01–11.44)	10.23 (7524.02)	3.31 (3.21)	10.02 (9.37)
	White blood cell count decreased	724	7.77 (7.21–8.37)	7.51 (4040.14)	2.87 (2.76)	7.40 (6.87)
	Haemoglobin decreased	651	8.00 (7.40–8.66)	7.76 (3786.50)	2.92 (2.80)	7.65 (7.07)
	Neutrophil count decreased	297	9.24 (8.23–10.37)	9.11 (2104.07)	3.12 (2.95)	8.94 (7.97)
	Blood count abnormal	277	9.97 (8.84–11.24)	9.84 (2155.04)	3.22 (3.04)	9.65 (8.56)
	Red blood cell count decreased	230	9.70 (8.50–11.06)	9.59 (1734.81)	3.18 (2.98)	9.41 (8.25)
	White blood cell count increased	184	6.31 (5.45–7.30)	6.26 (802.19)	2.58 (2.37)	6.18 (5.34)
	Laboratory test abnormal	140	4.09 (3.46–4.83)	4.06 (320.86)	1.97 (1.73)	4.03 (3.41)
	Haemoglobin abnormal	106	18.44 (15.18–22.41)	18.35 (1669.15)	3.92 (3.63)	17.65 (14.52)
	Blood lactate dehydrogenase increased*	93	8.48 (6.91–10.42)	8.45 (599.45)	2.93 (2.63)	8.31 (6.76)
	Blood test abnormal	85	6.50 (5.25–8.06)	6.48 (388.32)	2.57 (2.26)	6.40 (5.16)
	Lymphocyte count decreased	73	3.65 (2.9–4.59)	3.64 (138.53)	1.78 (1.44)	3.61 (2.87)
	White blood cell count abnormal	73	11.35 (9.00–14.33)	11.32 (669.38)	3.26 (2.92)	11.06 (8.76)
	Haematocrit decreased	71	4.89 (3.87–6.18)	4.87 (216.43)	2.18 (1.83)	4.83 (3.82)
	Blood potassium increased	69	5.10 (4.02–6.46)	5.08 (223.74)	2.23 (1.88)	5.03 (3.97)
	Full blood count decreased	69	3.48 (2.74–4.41)	3.47 (120.31)	1.72 (1.37)	3.45 (2.72)
	Blood potassium decreased	67	2.71 (2.13–3.45)	2.71 (71.67)	1.37 (1.02)	2.69 (2.12)
	Blood uric acid increased	67	15.79 (12.37–20.15)	15.74 (892.71)	3.63 (3.27)	15.22 (11.93)
	Platelet count abnormal	64	10.84 (8.46–13.9)	10.81 (556.10)	3.18 (2.82)	10.57 (8.25)
	Blood phosphorus increased*	59	27.96 (21.48–36.39)	27.88 (1437.59)	4.18 (3.80)	26.27 (20.18)
	Blood bilirubin increased	53	2.66 (2.03–3.48)	2.65 (54.25)	1.33 (0.93)	2.64 (2.02)
	Blood sodium decreased	41	2.91 (2.14–3.95)	2.90 (50.78)	1.43 (0.98)	2.89 (2.12)
	Full blood count abnormal	41	10.73 (7.87–14.63)	10.71 (352.41)	3.06 (2.60)	10.48 (7.68)
	Lymphocyte count increased	41	14.09 (10.32–19.23)	14.06 (481.96)	3.36 (2.90)	13.65 (10.00)
	Blast cell count increased*	32	32.02 (22.36–45.86)	31.97 (894.68)	3.95 (3.42)	29.86 (20.85)
	Neutrophil count abnormal	30	11.60 (8.07–16.68)	11.59 (282.72)	3.04 (2.50)	11.31 (7.87)
Metabolism and nutrition disorders	Tumour lysis syndrome	376	55.37 (49.69–61.69)	54.30 (17506.75)	5.42 (5.26)	48.41 (43.45)
	Hyperkalaemia	90	3.01 (2.45–3.71)	3.00 (119.62)	1.53 (1.23)	2.99 (2.43)
	Hypophagia	89	3.97 (3.22–4.89)	3.95 (194.66)	1.91 (1.60)	3.92 (3.18)
	Hyperuricaemia	63	20.62 (16.01–26.56)	20.56 (1119.73)	3.91 (3.53)	19.68 (15.28)
	Hypercalcaemia*	52	4.89 (3.72–6.43)	4.88 (158.66)	2.15 (1.74)	4.84 (3.68)
	Hypocalcaemia	51	3.14 (2.38–4.14)	3.14 (73.69)	1.56 (1.15)	3.12 (2.37)
	Hyperphosphataemia*	44	34.27 (25.21–46.6)	34.20 (1315.27)	4.21 (3.76)	31.79 (23.38)
	Fluid intake reduced	33	8.74 (6.19–12.34)	8.73 (221.50)	2.77 (2.26)	8.58 (6.08)
	Hypophosphataemia	31	5.00 (3.51–7.12)	4.99 (97.84)	2.09 (1.57)	4.95 (3.47)
Respiratory, thoracic and mediastinal disorders	Pleural effusion*	145	2.83 (2.40–3.33)	2.81 (169.06)	1.46 (1.22)	2.80 (2.38)

*Emerging findings of venetoclax associated AEs from FAERS database. SOC, system organ class; PT, preferred term; ROR, reporting odds ratio; CI, confidence interval; PRR, proportional reporting ratio; χ2, chi-information component; IC, information component; IC025, the lower limit of 95% CI of the IC; EBGM, empirical Bayesian geometric mean; EBGM05, the lower limit of 95% CI of EBGM.

### 3.3 Onset time of events

**Fig 1:**
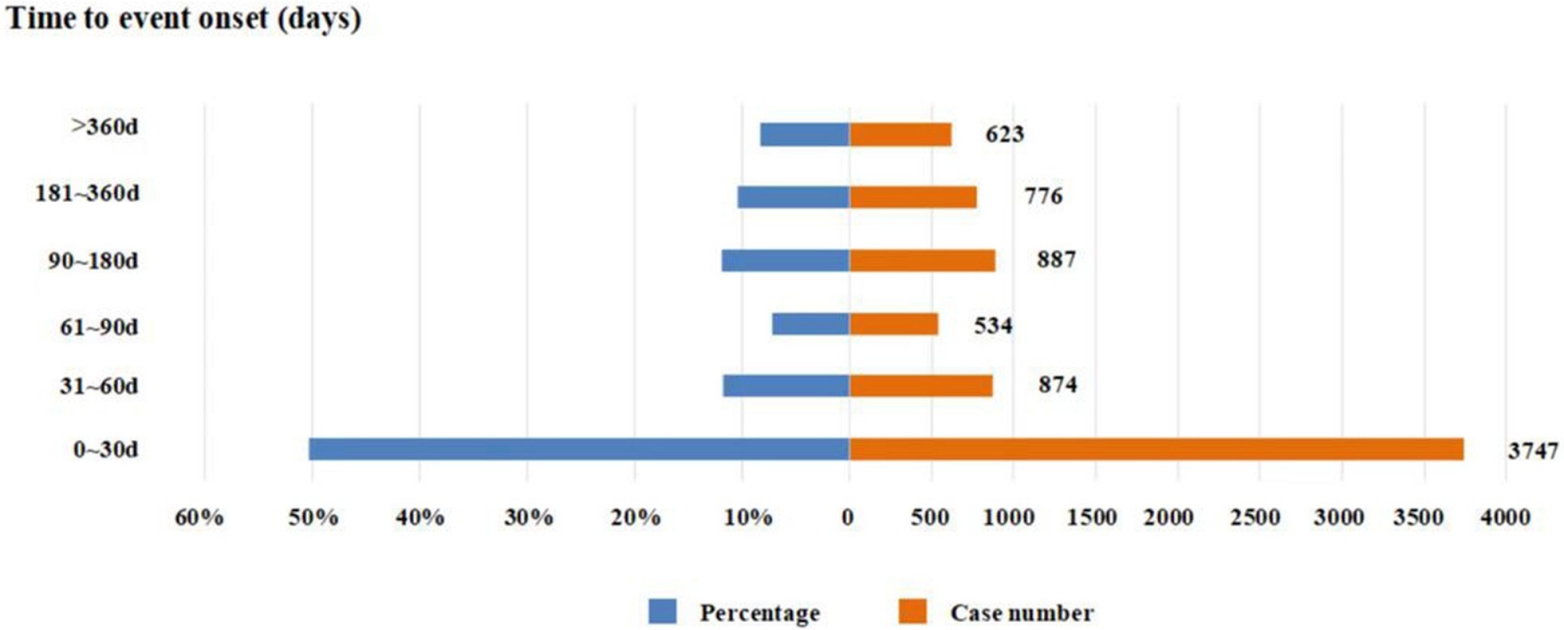
Time to onset of venetoclax-related AEs.

Excluding the unknown or unreported medication date or AEs occurrence date, the time to onset of AEs was calculated from the date obtained from the database. A total of 7,741 time to onset of AEs were obtained, with a median of 31 (7, 131) days. As shown in **Fig 1**, half of AEs occurred in the first month (n = 3,747, 50.36%), the second month (n = 874, 11.75%) and the third month (n = 534, 7.18%), and then gradually decreased.

## 4. Discussion

Most of the efficacy and safety data of newly marketed drugs come from clinical trials. However, due to the relatively small in sample size and strict in trial design, implementation and data analysis, clinical trials for registration cannot fully expose the impact of drugs on the human body in the real world, especially in terms of safety [16]. FAERS is a spontaneous reporting system for post-marketing drug safety monitoring that is open to the public [13,17]. Through data mining FAERS, the new and rare AEs can be evaluated and detected. Compared with the previous clinical studies on VEN [18–21], this study was based on a large sample size and a long time span, and found new AE signals, filling the gap in the study of VEN related AEs in the real world. It is of great significance to understand the safety of VEN. Moreover, this study excavated the occurrence time of VEN related AEs, providingthe reference for security of drug usage in clinical practice.

In western developed countries, CLL and AML are primarily diseases of older adults. In diagnosis of CLL and AML, the median age is 72 and 68 years old respectively, and the males are 1.7 and 1.6 times more likely than females, respectively [22–26]. From FAERS database, this study collected 19,107 AEs reports from 77 different countries and regions since the approval of VEN from 2016 to 2021. The clinical characteristics analysis in our study are roughly consistent with the approved indications and target population characteristics of VEN. The results of disproportionality analysis showed that at the SOC level, VEN-related AEs reported significant signals including general disorders and administration site conditions, infections and infestations, blood and lymphatic system disorders, investigations, respiratory, thoracic and mediastinal disorders. Significant AEs included neutropenia, thrombocytopenia, infection, tumour lysis syndrome, pneumonia, and sepsis. The above is consistent with the description of adverse reactions in the label of VEN.

The most common known AE of VEN on the human immune system is neutropenia, and no other obvious AEs have been seen [27]. A clinical trial of high-dose VEN (800 mg/day) monotherapy in 32 patients with relapsed or refractory AML or unfit for intensive chemotherapy reported that the serious AEs were febrile neutropenia (28%) and pneumonia (16%) [28]. The VIALE-A trial [29] showed the grade 3 or higher hematologic AEs reported in the VEN plus azacitidine group and the azacitidine alone group included thrombocytopenia (45% vs 38%), neutropenia (42% vs 28%), and febrile neutropenia (42% vs 19%). Our results found that among immune-related AEs, neutropenia occurred the most (768/19,107), which is consistent with the results reported above.

Due to immunosuppressive effects, infections are common AEs in use of VEN [30]. In CLL patients, infections are the major cause of morbidity and mortality, of which bacterial infections are the most common, affecting mainly the respiratory tract [31–34]. A study [35] showed that the incidence of invasive pulmonary aspergillosis was significantly higher in patients with haematologic malignancies than in patients with solid tumors, and higher in men than in women. Neutropenia has been reported in 40 to 50 percent of VEN users in several clinical trials, and serious infections have occurred in 15 percent of patients with grade ≥3 neutropenia [30,36,37]. Consistent with our findings, infection accounted for 23.3% (4,458/19,107) of VEN-related AEs, the most common of which included Pneumonia, Infection, and Sepsis, and all infection types included bacteria, fungi, and viruses. Notably, pneumonia was the largest type of infection, and although fungal pneumonia was limited in number of cases (64/19,107), it was strongly associated with VEN (ROR 22.86, PRR 22.79). Patients with AML are at a relatively higher risk of developing infections due to frequent baseline neutropenia and the potentiation of cytotoxic chemotherapeutics with VEN [38]. In the VIALE-A clinical trial of VEN for the treatment of AML, the proportion of participants in the VEN plus azacitidine group versus placebo plus azacitidine group was 85% and 67%, and the proportion of grade ≥3 was 64% and 51% [29]. The above results suggest that clinicians or pharmacists should monitor blood routines and pay close attention to infection indicators when patients use VEN. For patients with existing infection indications, it is necessary to weigh the pros and cons of continuing to use VEN, and adjust the dose or stop using it in time. However, a clinical trial in AML indicated that posaconazole should be used in antifungal prophylaxis with a VEN dose reduction of 75 percent [27,39].

As the first highly selective Bcl-2 inhibitor for clinical use, VEN has an over three orders of magnitude affinity for Bcl-2 than Bcl- X_L_ and Bcl-W [10], which can greatly improve the problem of dose-dependent thrombocytopenia caused by low selective Bcl-2 inhibitors acting on Bcl- X_L_ [7,40,41]. The above was further confirmed in this study. We found a low incidence of VEN-related thrombocytopenia in 373 cases (373/19,107) among all AEs reported, and this may be confounded with the role of other combination chemotherapeutic agents. The incidence of thrombocytopenia in a phase 2 clinical trial of VEN alone for R/R CLL was 15% [18]. While, a trial of VEN plus LDAC for patients with newly diagnosed AML ineligible for intensive chemotherapy showed that combination chemotherapy increased the risk of thrombocytopenia [19]. Attention to thrombocytopenia in VEN therapy, especially in combination chemotherapy, is still needed.

AML has the highest mortality rate in leukemia, accounting for 62%, especially for patients aged ≥ 65, nearly 80% will die within one year after diagnosis [25,26], and the proportion of AML among the VEN medication indications in our study reached 33.05%. In this study, the proportion of deaths (22.9%, 4,377/19,107) in all AEs is consistent with the above situation. Our research results also show that off-label use accounts for 12.2% (2,339/19,107) of the total number of reports, which indicates that off-label use seems to be very common in clinical use of VEN. The AML is the most indication, accounting for more than 1/3, CLL is close to 1/3, and the remaining 1/3 are currently unapproved indications [20,21,42]. Although off-label use gives patients hope and some efficacy, it also brings a higher risk of AEs than conventional use, which requires careful evaluation and treatment by clinicians [43,44].

The risk of developing tumor lysis syndrome (TLS) with VEN use is higher due to the strong and rapid efficacy of the drug [45,46]. A clinical study investigated the occurrence of TLS in 297 patients with CLL. The reported incidence of Clinical TLS was 2.7%, while the incidence of laboratory TLS was 5.7% [47]. In our study, the proportion of TLS in all AEs associate with VEN was 2% (376/19,107). The occurrence of TLS during drug use is related to tumor mass, comorbidities (especially renal function), and treatment dose. Therefore, assessing the risk of TLS before VEN administration by the criterion given in the label and monitoring creatinine clearance are effective measures to predict TLS. According to the patient’s condition, timely laboratory monitoring, use of uric acid-lowering drugs and adequate hydration are necessary approaches to prevent TLS [45,46].

By mining the FAERS database and analyzing the AE signals, this study explored the statistical link between VEN use and AEs in patients. However, this study still has limitations, on the one hand, FAERS database is a spontaneous reporting system, due to its own limitations, there are phenomena such as underreporting, re-reporting, incomplete case information and so on, and lack of underlying disease and concomitant medication may affect the results. On the other hand, voluntary reports are not restricted to health care professionals, and consumers are also candidates to report AES, whereas the medical expertise of the consumer is limited, unfortunately. Nonetheless, the findings of relevant AEs based on the FAERS database are still significant. Not only can it provide more evidence for the safe clinical use of VEN, but also some newly discovered signals of unexpected AEs can arouse people’s vigilance.

## 5. Conclusion

Based on the FAERS database, the disproportionality analysis was used to analyze the AEs of VEN after approval, and then the safety information of VEN in the real world was mined. The AEs excavated in this study were basically consistent with the AEs in the label, and AEs not mentioned in the label were also found, such as pleural effusion, atrial fibrillation, splenomegaly, spleen atrophy, and splenic infarction. The discovery of these strong signal AEs, to a certain extent, can supplement the relatively small sample size of premarket clinical research. We still need to further study the correlation between the novelty VEN-related AEs and the drug itself, and perfect the safety information of VEN. Only by fully understanding the AEs of VEN can it help to avoid the risks of use, identify the types of AEs in a timely manner and deal with them correctly, and ultimately improve the effect of drug treatment and achieve the goal of optimal treatment for patients.

## Supporting information

S1 Table Four major algorithms used for signal detection. (DOCX)

S2 Table Signal strength of all AEs reports associated with venetoclax at the PT level. (DOCX)
