# Supplementary material for: A real-world pharmacovigilance study of FDA Adverse Event Reporting System (FAERS) events for venetoclax
Source: PLoS One. 2022 Dec 7;17(12):e0278725. doi: 10.1371/journal.pone.0278725 (PMC9728853; doi:10.1371/journal.pone.0278725)
Supplement: S2 Table — (DOCX) [file pone.0278725.s002.docx]

**S2 Table.** Signal strength of all AEs reports associated with Venetoclax at the PT level.

| **SOC** | **PT** | **Case number**  **(n)** | **ROR**  **(95% two-sided CI)** | **PRR**  **(χ^2^)** | **IC**  **(IC025)** | **EBGM**  **(EBGM05)** |
| --- | --- | --- | --- | --- | --- | --- |
| Blood and lymphatic system disorders | Neutropenia | 768 | 7.05 (6.56-7.59) | 6.81 (3771.94) | 2.74 (2.63) | 6.72 (6.25) |
|  | Febrile neutropenia | 553 | 10.31 (9.47-11.23) | 10.04 (4414.75) | 3.27 (3.15) | 9.84 (9.03) |
|  | Thrombocytopenia | 373 | 4.07 (3.67-4.51) | 4.01 (839.08) | 1.98 (1.83) | 3.98 (3.59) |
|  | Pancytopenia | 341 | 7.89 (7.08-8.79) | 7.76 (1979.06) | 2.90 (2.74) | 7.65 (6.86) |
|  | Lymphadenopathy | 262 | 9.24 (8.17-10.45) | 9.13 (1860.59) | 3.12 (2.93) | 8.96 (7.92) |
|  | Cytopenia | 150 | 14.57 (12.38-17.16) | 14.47 (1821.2) | 3.68 (3.44) | 14.04 (11.92) |
|  | Splenomegaly | 94 | 9.07 (7.39-11.13) | 9.03 (658.19) | 3.02 (2.72) | 8.87 (7.23) |
|  | Bone marrow failure | 88 | 3.69 (2.99-4.55) | 3.68 (170.27) | 1.81 (1.5) | 3.65 (2.96) |
|  | Myelosuppression | 79 | 6.65 (5.32-8.31) | 6.63 (372.17) | 2.60 (2.27) | 6.54 (5.24) |
|  | Autoimmune haemolyticanaemia | 65 | 18.31 (14.28-23.48) | 18.26 (1017.90) | 3.79 (3.42) | 17.56 (13.70) |
|  | Platelet disorder | 43 | 19.26 (14.19-26.15) | 19.22 (711.49) | 3.69 (3.24) | 18.45 (13.59) |
|  | Haemolyticanaemia | 38 | 5.31 (3.86-7.32) | 5.30 (131.17) | 2.21 (1.73) | 5.25 (3.81) |
|  | Bone marrow disorder | 36 | 16.10 (11.54-22.46) | 16.07 (490.93) | 3.44 (2.95) | 15.54 (11.14) |
|  | Haemolysis | 34 | 5.02 (3.58-7.04) | 5.01 (108.01) | 2.12 (1.62) | 4.97 (3.54) |
|  | Blood disorder | 31 | 4.72 (3.31-6.72) | 4.71 (89.66) | 2.02 (1.50) | 4.67 (3.28) |
|  | Haematotoxicity | 29 | 3.71 (2.57-5.35) | 3.7 (56.78) | 1.71 (1.17) | 3.68 (2.55) |
|  | Immune thrombocytopenia | 23 | 7.64 (5.06-11.55) | 7.64 (130.38) | 2.50 (1.90) | 7.52 (4.98) |
|  | Lymphocytosis | 23 | 13.02 (8.6-19.71) | 13.00 (247.50) | 3.03 (2.42) | 12.66 (8.36) |
|  | White blood cell disorder | 22 | 8.61 (5.65-13.14) | 8.60 (144.99) | 2.61 (1.99) | 8.46 (5.54) |
|  | Febrile bone marrow aplasia | 18 | 4.40 (2.76-6.99) | 4.39 (46.71) | 1.81 (1.13) | 4.36 (2.74) |
|  | Aplastic anaemia | 15 | 3.82 (2.29-6.34) | 3.81 (30.88) | 1.60 (0.85) | 3.79 (2.28) |
|  | Abdominal lymphadenopathy | 10 | 10.55 (5.63-19.75) | 10.54 (84.36) | 2.34 (1.42) | 10.32 (5.51) |
|  | Aplasia pure red cell | 10 | 4.80 (2.57-8.95) | 4.80 (29.74) | 1.69 (0.77) | 4.76 (2.55) |
|  | Splenic infarction | 10 | 6.78 (3.63-12.66) | 6.77 (48.46) | 2.00 (1.08) | 6.69 (3.58) |
|  | Red blood cell abnormality | 9 | 10.53 (5.44-20.41) | 10.53 (75.8) | 2.26 (1.29) | 10.31 (5.32) |
|  | Lymph node pain | 8 | 5.32 (2.65-10.69) | 5.32 (27.73) | 1.67 (0.64) | 5.27 (2.62) |
|  | Spleen disorder | 8 | 5.23 (2.6-10.5) | 5.22 (27.01) | 1.65 (0.63) | 5.17 (2.58) |
|  | Bone marrow infiltration | 5 | 10.52 (4.33-25.55) | 10.52 (42.06) | 1.75 (0.45) | 10.29 (4.24) |
|  | Lymphatic disorder | 5 | 9.64 (3.97-23.39) | 9.64 (37.88) | 1.71 (0.40) | 9.45 (3.90) |
|  | Anisocytosis | 4 | 8.93 (3.32-24.04) | 8.93 (27.60) | 1.46 (0.00) | 8.77 (3.26) |
|  | Reticulocytosis | 4 | 20.36 (7.47-55.48) | 20.35 (70.33) | 1.73 (0.26) | 19.49 (7.15) |
|  | Spleen atrophy | 4 | 62.52 (21.93-178.27) | 62.51 (211.84) | 1.90 (0.37) | 54.82 (19.23) |
| Cardiac disorders | Atrial fibrillation | 238 | 2.84 (2.50-3.23) | 2.82 (279.12) | 1.47 (1.28) | 2.81 (2.47) |
| Congenital, familial and genetic disorders | Aplasia | 19 | 8.47 (5.38-13.35) | 8.47 (122.74) | 2.53 (1.86) | 8.32 (5.29) |
| Gastrointestinal disorders | Intra-abdominal fluid collection | 13 | 5.48 (3.17-9.48) | 5.48 (47.03) | 1.94 (1.13) | 5.42 (3.14) |
|  | Oesophageal stenosis | 10 | 4.67 (2.50-8.70) | 4.66 (28.49) | 1.66 (0.74) | 4.63 (2.48) |
|  | Neutropenic colitis | 9 | 5.07 (2.63-9.78) | 5.07 (29.06) | 1.69 (0.72) | 5.02 (2.60) |
| General disorders and administration site conditions | Death | 4377 | 6.84 (6.61-7.08) | 5.50 (16632.74) | 2.44 (2.40) | 5.45 (5.27) |
|  | Pyrexia | 846 | 2.99 (2.79-3.20) | 2.90 (1062.76) | 1.53 (1.42) | 2.89 (2.69) |
|  | Disease progression | 346 | 4.04 (3.63-4.49) | 3.98 (769.64) | 1.97 (1.81) | 3.96 (3.56) |
|  | Decreased activity | 65 | 7.49 (5.86-9.57) | 7.47 (358.00) | 2.72 (2.36) | 7.36 (5.75) |
|  | Adverse food reaction | 10 | 12.65 (6.75-23.73) | 12.65 (104.25) | 2.46 (1.54) | 12.32 (6.57) |
|  | Multimorbidity | 8 | 9.46 (4.70-19.07) | 9.46 (59.25) | 2.10 (1.07) | 9.28 (4.61) |
|  | Critical illness | 7 | 8.19 (3.88-17.31) | 8.19 (43.37) | 1.91 (0.80) | 8.06 (3.81) |
|  | Medical device site haemorrhage | 4 | 11.29 (4.19-30.48) | 11.29 (36.58) | 1.55 (0.09) | 11.03 (4.09) |
| Hepatobiliary disorders | Hepatic lesion | 14 | 3.86 (2.28-6.53) | 3.86 (29.35) | 1.59 (0.81) | 3.83 (2.26) |
|  | Hepatosplenomegaly | 9 | 4.92 (2.55-9.49) | 4.92 (27.77) | 1.66 (0.69) | 4.87 (2.53) |
| Immune system disorders | Immunodeficiency | 82 | 5.79 (4.66-7.21) | 5.77 (319.49) | 2.42 (2.09) | 5.71 (4.59) |
|  | Hypogammaglobulinaemia | 24 | 4.63 (3.10-6.93) | 4.63 (67.52) | 1.95 (1.35) | 4.59 (3.07) |
|  | Cytokine storm | 6 | 10.34 (4.6-23.23) | 10.34 (49.43) | 1.91 (0.72) | 10.12 (4.50) |
| Infections and infestations | Pneumonia | 957 | 3.36 (3.15-3.58) | 3.24 (1494.27) | 1.68 (1.59) | 3.22 (3.02) |
|  | Infection | 529 | 4.21 (3.86-4.6) | 4.12 (1248.74) | 2.02 (1.90) | 4.10 (3.75) |
|  | Sepsis | 488 | 5.05 (4.61-5.53) | 4.94 (1526.40) | 2.28 (2.15) | 4.90 (4.48) |
|  | COVID-19 | 322 | 3.61 (3.23-4.03) | 3.57 (593.20) | 1.81 (1.65) | 3.55 (3.18) |
|  | Septic shock | 142 | 3.84 (3.26-4.54) | 3.82 (293.80) | 1.89 (1.64) | 3.80 (3.22) |
|  | Fungal infection | 81 | 2.69 (2.16-3.35) | 2.68 (85.26) | 1.37 (1.05) | 2.67 (2.15) |
|  | Diverticulitis | 71 | 2.72 (2.16-3.44) | 2.72 (76.70) | 1.38 (1.04) | 2.71 (2.14) |
|  | Clostridium difficile infection | 70 | 2.97 (2.35-3.76) | 2.97 (90.79) | 1.50 (1.16) | 2.95 (2.33) |
|  | COVID-19 pneumonia | 70 | 6.89 (5.44-8.72) | 6.86 (345.49) | 2.63 (2.28) | 6.77 (5.35) |
|  | Pneumonia fungal | 64 | 22.86 (17.77-29.40) | 22.79 (1267.33) | 4.02 (3.65) | 21.71 (16.88) |
|  | Localised infection | 63 | 2.73 (2.13-3.50) | 2.73 (68.48) | 1.38 (1.01) | 2.71 (2.12) |
|  | Bacteraemia | 61 | 6.14 (4.77-7.91) | 6.13 (258.17) | 2.46 (2.09) | 6.06 (4.70) |
|  | Bacterial infection | 50 | 3.38 (2.55-4.46) | 3.37 (82.74) | 1.65 (1.24) | 3.35 (2.54) |
|  | Skin infection | 43 | 4.17 (3.08-5.63) | 4.16 (102.22) | 1.91 (1.47) | 4.13 (3.06) |
|  | Neutropenic sepsis | 42 | 6.85 (5.05-9.30) | 6.84 (206.24) | 2.54 (2.09) | 6.75 (4.97) |
|  | Pneumocystis jirovecii pneumonia | 35 | 3.28 (2.35-4.57) | 3.27 (54.81) | 1.57 (1.08) | 3.25 (2.33) |
|  | Bronchopulmonary aspergillosis | 34 | 4.99 (3.55-6.99) | 4.98 (106.94) | 2.11 (1.61) | 4.93 (3.52) |
|  | Aspergillus infection | 33 | 4.71 (3.34-6.63) | 4.70 (95.15) | 2.03 (1.52) | 4.66 (3.31) |
|  | Escherichia infection | 33 | 4.79 (3.40-6.75) | 4.78 (97.66) | 2.05 (1.55) | 4.74 (3.36) |
|  | Vascular device infection | 32 | 8.43 (5.94-11.97) | 8.42 (205.33) | 2.72 (2.20) | 8.28 (5.83) |
|  | Bacterial sepsis | 30 | 11.41 (7.94-16.4) | 11.39 (277.30) | 3.02 (2.49) | 11.13 (7.74) |
|  | Escherichia bacteraemia | 25 | 12.59 (8.46-18.74) | 12.57 (258.92) | 3.04 (2.45) | 12.25 (8.23) |
|  | Rhinovirus infection | 25 | 7.41 (4.99-11.01) | 7.41 (136.25) | 2.50 (1.92) | 7.30 (4.91) |
|  | Soft tissue infection | 23 | 15.77 (10.40-23.91) | 15.75 (306.69) | 3.20 (2.58) | 15.24 (10.05) |
|  | Escherichia sepsis | 22 | 9.11 (5.97-13.90) | 9.10 (155.38) | 2.67 (2.05) | 8.93 (5.86) |
|  | Pseudomonas infection | 22 | 3.20 (2.10-4.87) | 3.20 (32.99) | 1.47 (0.86) | 3.18 (2.09) |
|  | Pseudomonal sepsis | 17 | 12.53 (7.74-20.3) | 12.52 (175.25) | 2.83 (2.12) | 12.20 (7.53) |
|  | Staphylococcal bacteraemia | 17 | 5.18 (3.21-8.36) | 5.18 (56.68) | 1.98 (1.27) | 5.13 (3.18) |
|  | Pneumonia viral | 15 | 5.04 (3.03-8.38) | 5.03 (47.94) | 1.90 (1.15) | 4.99 (3.00) |
|  | Device related sepsis | 13 | 6.58 (3.80-11.38) | 6.58 (60.56) | 2.11 (1.31) | 6.49 (3.75) |
|  | Staphylococcal sepsis | 13 | 3.68 (2.13-6.35) | 3.67 (25.10) | 1.51 (0.71) | 3.65 (2.12) |
|  | Pneumonia pseudomonal | 11 | 7.80 (4.30-14.17) | 7.80 (64.08) | 2.18 (1.30) | 7.68 (4.23) |
|  | Klebsiella sepsis | 10 | 11.11 (5.93-20.82) | 11.11 (89.69) | 2.38 (1.46) | 10.86 (5.79) |
|  | Pseudomonal bacteraemia | 10 | 14.59 (7.77-27.41) | 14.59 (122.46) | 2.55 (1.62) | 14.15 (7.53) |
|  | Sinusitis fungal | 10 | 15.58 (8.29-29.28) | 15.57 (131.68) | 2.59 (1.66) | 15.07 (8.02) |
|  | Enterococcal bacteraemia | 9 | 11.52 (5.94-22.33) | 11.51 (84.20) | 2.32 (1.35) | 11.25 (5.80) |
|  | Febrile infection | 9 | 8.83 (4.57-17.09) | 8.83 (61.25) | 2.14 (1.17) | 8.67 (4.48) |
|  | Fungal sepsis | 9 | 12.71 (6.55-24.66) | 12.70 (94.31) | 2.38 (1.41) | 12.37 (6.38) |
|  | Neutropenic infection | 9 | 16.15 (8.30-31.41) | 16.14 (123.27) | 2.51 (1.53) | 15.60 (8.02) |
|  | Streptococcal bacteraemia | 9 | 10.51 (5.42-20.35) | 10.50 (75.56) | 2.26 (1.29) | 10.28 (5.31) |
|  | Campylobacter gastroenteritis | 8 | 13.90 (6.87-28.1) | 13.89 (92.76) | 2.33 (1.29) | 13.49 (6.67) |
|  | Medical device site infection | 8 | 11.33 (5.62-22.87) | 11.33 (73.44) | 2.22 (1.18) | 11.07 (5.49) |
|  | Parainfluenzae virus infection | 8 | 5.00 (2.49-10.04) | 5.00 (25.32) | 1.61 (0.59) | 4.96 (2.47) |
|  | Pneumonia influenzal | 8 | 6.76 (3.36-13.59) | 6.76 (38.65) | 1.86 (0.83) | 6.67 (3.32) |
|  | Pneumonia klebsiella | 8 | 5.32 (2.65-10.69) | 5.32 (27.73) | 1.67 (0.64) | 5.27 (2.62) |
|  | Pulmonary sepsis | 7 | 4.87 (2.31-10.26) | 4.87 (21.29) | 1.51 (0.42) | 4.83 (2.29) |
|  | Campylobacter infection | 6 | 8.99 (4.01-20.19) | 8.99 (41.76) | 1.84 (0.65) | 8.83 (3.93) |
|  | Epididymitis | 6 | 6.01 (2.68-13.45) | 6.01 (24.71) | 1.58 (0.39) | 5.94 (2.65) |
|  | Lower respiratory tract infection fungal | 6 | 13.33 (5.92-30.04) | 13.33 (66.39) | 2.04 (0.84) | 12.96 (5.75) |
|  | Pneumocystis jirovecii infection | 6 | 5.72 (2.56-12.80) | 5.72 (23.07) | 1.54 (0.36) | 5.66 (2.53) |
|  | Sinusitis bacterial | 6 | 8.21 (3.66-18.41) | 8.20 (37.26) | 1.78 (0.59) | 8.07 (3.60) |
|  | Candida sepsis | 5 | 7.10 (2.94-17.19) | 7.10 (25.80) | 1.54 (0.24) | 7.01 (2.90) |
|  | Clostridium colitis | 5 | 12.87 (5.29-31.33) | 12.87 (53.17) | 1.84 (0.53) | 12.53 (5.15) |
|  | Lymph gland infection | 5 | 12.36 (5.08-30.08) | 12.36 (50.77) | 1.82 (0.51) | 12.05 (4.95) |
|  | Streptococcal sepsis | 5 | 5.88 (2.43-14.22) | 5.88 (19.99) | 1.43 (0.13) | 5.82 (2.41) |
|  | Herpes simplex reactivation | 4 | 21.09 (7.73-57.53) | 21.09 (73.02) | 1.74 (0.26) | 20.16 (7.39) |
|  | Sinusitis aspergillus | 4 | 25.74 (9.39-70.58) | 25.74 (89.83) | 1.78 (0.30) | 24.36 (8.89) |
|  | Spinal cord abscess | 4 | 12.33 (4.56-33.30) | 12.33 (40.49) | 1.59 (0.12) | 12.02 (4.45) |
| Injury, poisoning and procedural complications | Off label use | 2339 | 3.03 (2.91-3.17) | 2.79 (2782.97) | 1.47 (1.41) | 2.77 (2.66) |
|  | Intentional product use issue | 647 | 6.65 (6.15-7.20) | 6.46 (2958.43) | 2.66 (2.54) | 6.38 (5.90) |
|  | Transfusion reaction | 12 | 20.60 (11.55-36.77) | 20.59 (213.63) | 2.90 (2.05) | 19.71 (11.05) |
|  | Intentional device use issue | 7 | 11.35 (5.36-24.04) | 11.34 (64.36) | 2.10 (1.00) | 11.08 (5.23) |
|  | Animal scratch | 6 | 7.46 (3.33-16.72) | 7.46 (32.99) | 1.72 (0.54) | 7.35 (3.28) |
|  | Systemic toxicity | 6 | 38.62 (16.76-89) | 38.61 (201.99) | 2.36 (1.14) | 35.56 (15.43) |
|  | Transplantation complication | 6 | 13.75 (6.10-30.99) | 13.75 (68.75) | 2.05 (0.85) | 13.36 (5.93) |
|  | Complications of transplant surgery | 5 | 19.89 (8.12-48.75) | 19.89 (85.80) | 1.99 (0.67) | 19.07 (7.78) |
|  | Post procedural contusion | 4 | 12.69 (4.69-34.28) | 12.68 (41.84) | 1.60 (0.13) | 12.35 (4.57) |
| Investigations | Platelet count decreased | 920 | 10.70 (10.01-11.44) | 10.23 (7524.02) | 3.31 (3.21) | 10.02 (9.37) |
|  | White blood cell count decreased | 724 | 7.77 (7.21-8.37) | 7.51 (4040.14) | 2.87 (2.76) | 7.40 (6.87) |
|  | Haemoglobin decreased | 651 | 8.00 (7.40-8.66) | 7.76 (3786.50) | 2.92 (2.80) | 7.65 (7.07) |
|  | Neutrophil count decreased | 297 | 9.24 (8.23-10.37) | 9.11 (2104.07) | 3.12 (2.95) | 8.94 (7.97) |
|  | Blood count abnormal | 277 | 9.97 (8.84-11.24) | 9.84 (2155.04) | 3.22 (3.04) | 9.65 (8.56) |
|  | Red blood cell count decreased | 230 | 9.70 (8.50-11.06) | 9.59 (1734.81) | 3.18 (2.98) | 9.41 (8.25) |
|  | White blood cell count increased | 184 | 6.31 (5.45-7.30) | 6.26 (802.19) | 2.58 (2.37) | 6.18 (5.34) |
|  | Laboratory test abnormal | 140 | 4.09 (3.46-4.83) | 4.06 (320.86) | 1.97 (1.73) | 4.03 (3.41) |
|  | Haemoglobin abnormal | 106 | 18.44 (15.18-22.41) | 18.35 (1669.15) | 3.92 (3.63) | 17.65 (14.52) |
|  | Blood lactate dehydrogenase increased | 93 | 8.48 (6.91-10.42) | 8.45 (599.45) | 2.93 (2.63) | 8.31 (6.76) |
|  | Blood test abnormal | 85 | 6.50 (5.25-8.06) | 6.48 (388.32) | 2.57 (2.26) | 6.40 (5.16) |
|  | Lymphocyte count decreased | 73 | 3.65 (2.90-4.59) | 3.64 (138.53) | 1.78 (1.44) | 3.61 (2.87) |
|  | White blood cell count abnormal | 73 | 11.35 (9.00-14.33) | 11.32 (669.38) | 3.26 (2.92) | 11.06 (8.76) |
|  | Haematocrit decreased | 71 | 4.89 (3.87-6.18) | 4.87 (216.43) | 2.18 (1.83) | 4.83 (3.82) |
|  | Blood potassium increased | 69 | 5.10 (4.02-6.46) | 5.08 (223.74) | 2.23 (1.88) | 5.03 (3.97) |
|  | Full blood count decreased | 69 | 3.48 (2.74-4.41) | 3.47 (120.31) | 1.72 (1.37) | 3.45 (2.72) |
|  | Blood potassium decreased | 67 | 2.71 (2.13-3.45) | 2.71 (71.67) | 1.37 (1.02) | 2.69 (2.12) |
|  | Blood uric acid increased | 67 | 15.79 (12.37-20.15) | 15.74 (892.71) | 3.63 (3.27) | 15.22 (11.93) |
|  | Platelet count abnormal | 64 | 10.84 (8.46-13.9) | 10.81 (556.10) | 3.18 (2.82) | 10.57 (8.25) |
|  | Blood phosphorus increased | 59 | 27.96 (21.48-36.39) | 27.88 (1437.59) | 4.18 (3.80) | 26.27 (20.18) |
|  | Blood bilirubin increased | 53 | 2.66 (2.03-3.48) | 2.65 (54.25) | 1.33 (0.93) | 2.64 (2.02) |
|  | Blood sodium decreased | 41 | 2.91 (2.14-3.95) | 2.90 (50.78) | 1.43 (0.98) | 2.89 (2.12) |
|  | Full blood count abnormal | 41 | 10.73 (7.87-14.63) | 10.71 (352.41) | 3.06 (2.60) | 10.48 (7.68) |
|  | Lymphocyte count increased | 41 | 14.09 (10.32-19.23) | 14.06 (481.96) | 3.36 (2.90) | 13.65 (10.00) |
|  | Blast cell count increased | 32 | 32.02 (22.36-45.86) | 31.97 (894.68) | 3.95 (3.42) | 29.86 (20.85) |
|  | Neutrophil count abnormal | 30 | 11.60 (8.07-16.68) | 11.59 (282.72) | 3.04 (2.50) | 11.31 (7.87) |
|  | Blood magnesium decreased | 29 | 3.77 (2.61-5.43) | 3.76 (58.36) | 1.73 (1.19) | 3.74 (2.59) |
|  | Blood calcium increased | 24 | 3.72 (2.49-5.56) | 3.72 (47.29) | 1.68 (1.09) | 3.69 (2.47) |
|  | General physical condition abnormal | 23 | 3.54 (2.35-5.34) | 3.54 (41.52) | 1.61 (1.00) | 3.52 (2.33) |
|  | Red blood cell count abnormal | 21 | 15.18 (9.82-23.46) | 15.16 (268.53) | 3.11 (2.47) | 14.69 (9.50) |
|  | Blood creatinine abnormal | 19 | 5.81 (3.69-9.13) | 5.80 (74.53) | 2.14 (1.47) | 5.74 (3.65) |
|  | Investigation abnormal | 15 | 14.13 (8.45-23.63) | 14.12 (177.09) | 2.84 (2.08) | 13.71 (8.19) |
|  | Protein total decreased | 15 | 5.55 (3.34-9.24) | 5.55 (55.23) | 2.01 (1.26) | 5.49 (3.30) |
|  | Biopsy bone marrow | 12 | 43.07 (23.80-77.93) | 43.04 (448.63) | 3.2 (2.33) | 39.27 (21.70) |
|  | Blood immunoglobulin G decreased | 12 | 7.05 (3.99-12.48) | 7.05 (61.30) | 2.14 (1.30) | 6.95 (3.93) |
|  | Blood potassium abnormal | 12 | 5.04 (2.85-8.91) | 5.04 (38.41) | 1.82 (0.98) | 4.99 (2.83) |
|  | Blood uric acid abnormal | 12 | 45.29 (24.99-82.08) | 45.27 (470.76) | 3.22 (2.34) | 41.12 (22.69) |
|  | Immunoglobulins decreased | 12 | 10.90 (6.15-19.33) | 10.89 (105.21) | 2.50 (1.65) | 10.65 (6.01) |
|  | Blast cells present | 10 | 19.63 (10.42-37.00) | 19.62 (169.15) | 2.71 (1.78) | 18.82 (9.99) |
|  | Red blood cell count increased | 10 | 3.76 (2.02-7.02) | 3.76 (20.11) | 1.44 (0.53) | 3.74 (2.01) |
|  | Biopsy bone marrow abnormal | 8 | 46.69 (22.52-96.81) | 46.67 (323.10) | 2.75 (1.68) | 42.27 (20.39) |
|  | Blast cell count decreased | 7 | 113.48 (49.41-260.63) | 113.44 (619.54) | 2.70 (1.50) | 90.29 (39.32) |
|  | Monocyte count decreased | 7 | 5.73 (2.72-12.07) | 5.73 (26.95) | 1.65 (0.55) | 5.66 (2.69) |
|  | Myocardial necrosis marker increased | 7 | 4.45 (2.11-9.38) | 4.45 (18.55) | 1.44 (0.34) | 4.42 (2.10) |
|  | Blast cells | 6 | 138.22 (55.20-346.13) | 138.18 (621.01) | 2.50 (1.19) | 105.26 (42.03) |
|  | Blood lactate dehydrogenase abnormal | 6 | 9.02 (4.02-20.26) | 9.02 (41.93) | 1.84 (0.65) | 8.86 (3.95) |
|  | Granulocyte count decreased | 6 | 7.84 (3.50-17.58) | 7.84 (35.16) | 1.76 (0.57) | 7.72 (3.44) |
|  | Myeloblast count increased | 6 | 105.05 (43.09-256.10) | 105.02 (498.51) | 2.49 (1.20) | 84.88 (34.82) |
|  | Haptoglobin decreased | 5 | 6.71 (2.77-16.24) | 6.71 (23.93) | 1.51 (0.21) | 6.62 (2.74) |
|  | Monoclonal immunoglobulin present | 5 | 6.90 (2.85-16.70) | 6.90 (24.84) | 1.53 (0.23) | 6.81 (2.81) |
|  | Aspiration bone marrow | 4 | 87.53 (29.92-256.12) | 87.51 (285.08) | 1.92 (0.36) | 73.09 (24.98) |
|  | Blood phosphorus abnormal | 4 | 10.06 (3.73-27.11) | 10.06 (31.90) | 1.51 (0.05) | 9.86 (3.66) |
|  | Granulocytes abnormal | 4 | 21.35 (7.82-58.25) | 21.34 (73.96) | 1.74 (0.27) | 20.40 (7.48) |
|  | Light chain analysis abnormal | 4 | 21.61 (7.92-58.98) | 21.61 (74.92) | 1.74 (0.27) | 20.64 (7.56) |
|  | SARS-CoV-2 antibody test negative | 4 | 10.94 (4.06-29.51) | 10.94 (35.24) | 1.54 (0.08) | 10.7 (3.97) |
| Metabolism and nutrition disorders | Tumour lysis syndrome | 376 | 55.37 (49.69-61.69) | 54.30 (17506.75) | 5.42 (5.26) | 48.41 (43.45) |
|  | Hyperkalaemia | 90 | 3.01 (2.45-3.71) | 3.00 (119.62) | 1.53 (1.23) | 2.99 (2.43) |
|  | Hypophagia | 89 | 3.97 (3.22-4.89) | 3.95 (194.66) | 1.91 (1.60) | 3.920 (3.18) |
|  | Hyperuricaemia | 63 | 20.62 (16.01-26.56) | 20.56 (1119.73) | 3.91 (3.53) | 19.68 (15.28) |
|  | Hypercalcaemia | 52 | 4.89 (3.72-6.43) | 4.88 (158.66) | 2.15 (1.74) | 4.84 (3.68) |
|  | Hypocalcaemia | 51 | 3.14 (2.38-4.14) | 3.14 (73.69) | 1.56 (1.15) | 3.12 (2.37) |
|  | Hyperphosphataemia | 44 | 34.27 (25.21-46.60) | 34.20 (1315.27) | 4.21 (3.76) | 31.79 (23.38) |
|  | Fluid intake reduced | 33 | 8.74 (6.19-12.34) | 8.73 (221.50) | 2.77 (2.26) | 8.58 (6.08) |
|  | Hypophosphataemia | 31 | 5.00 (3.51-7.12) | 4.99 (97.84) | 2.09 (1.57) | 4.95 (3.47) |
| Musculoskeletal and connective tissue disorders | Chondrocalcinosis pyrophosphate | 7 | 6.63 (3.14-13.99) | 6.63 (32.97) | 1.76 (0.66) | 6.55 (3.10) |
|  | Axillary mass | 6 | 6.93 (3.09-15.52) | 6.93 (29.96) | 1.68 (0.49) | 6.83 (3.05) |
| Neoplasms benign, malignant and unspecified (incl cysts and polyps) | Acute myeloid leukaemia | 597 | 53.06 (48.69-57.83) | 51.43 (26435.65) | 5.42 (5.29) | 46.13 (42.33) |
|  | Chronic lymphocytic leukaemia | 389 | 117.78 (105.23-131.82) | 115.40 (34916.76) | 6.21 (6.05) | 91.52 (81.78) |
|  | Richter's syndrome | 162 | 252.61 (208.14-306.58) | 250.48 (25601.30) | 6.33 (6.06) | 159.66 (131.55) |
|  | Leukaemia | 161 | 18.14 (15.49-21.25) | 18.00 (2483.87) | 3.97 (3.73) | 17.33 (14.79) |
|  | Myelodysplastic syndrome | 126 | 11.10 (9.30-13.26) | 11.04 (1122.29) | 3.31 (3.05) | 10.79 (9.04) |
|  | Acute myeloid leukaemia recurrent | 87 | 60.89 (48.64-76.23) | 60.62 (4480.90) | 5.05 (4.72) | 53.36 (42.63) |
|  | Lymphoma | 74 | 5.37 (4.27-6.76) | 5.36 (259.27) | 2.31 (1.97) | 5.30 (4.22) |
|  | Mantle cell lymphoma | 63 | 60.65 (46.59-78.96) | 60.45 (3236.65) | 4.85 (4.47) | 53.24 (40.89) |
|  | Neoplasm | 50 | 5.14 (3.89-6.80) | 5.13 (164.56) | 2.21 (1.80) | 5.09 (3.85) |
|  | Squamous cell carcinoma | 44 | 4.86 (3.61-6.54) | 4.85 (133.10) | 2.12 (1.68) | 4.81 (3.57) |
|  | Chronic lymphocytic leukaemia recurrent | 39 | 84.23 (59.78-118.70) | 84.06 (2685.19) | 4.65 (4.15) | 70.68 (50.16) |
|  | Diffuse large B-cell lymphoma | 37 | 5.66 (4.09-7.83) | 5.65 (139.94) | 2.28 (1.80) | 5.59 (4.04) |
|  | Squamous cell carcinoma of skin | 31 | 5.67 (3.98-8.08) | 5.66 (117.49) | 2.25 (1.72) | 5.60 (3.93) |
|  | Leukaemia recurrent | 24 | 18.38 (12.22-27.66) | 18.36 (378.10) | 3.35 (2.75) | 17.66 (11.74) |
|  | Non-Hodgkin's lymphoma | 24 | 4.14 (2.77-6.19) | 4.14 (56.55) | 1.81 (1.22) | 4.11 (2.75) |
|  | B-cell lymphoma | 21 | 6.56 (4.26-10.10) | 6.55 (97.40) | 2.31 (1.67) | 6.47 (4.21) |
|  | Recurrent cancer | 20 | 5.27 (3.39-8.19) | 5.26 (68.24) | 2.05 (1.40) | 5.21 (3.35) |
|  | Chronic lymphocytic leukaemia transformation | 18 | 254.31 (142.25-454.65) | 254.07 (2870.58) | 4.02 (3.22) | 161.11 (90.11) |
|  | Hodgkin's disease | 14 | 3.95 (2.33-6.69) | 3.95 (30.57) | 1.62 (0.84) | 3.92 (2.32) |
|  | Acute myeloid leukaemia refractory | 13 | 126.49 (68.23-234.51) | 126.41 (1254.89) | 3.52 (2.64) | 98.30 (53.02) |
|  | Chronic lymphocytic leukaemia refractory | 11 | 126.74 (64.77-247.97) | 126.66 (1063.53) | 3.31 (2.34) | 98.45 (50.32) |
|  | Minimal residual disease | 11 | 28.50 (15.48-52.45) | 28.48 (273.85) | 2.96 (2.07) | 26.80 (14.56) |
|  | T-cell prolymphocytic leukaemia | 11 | 166.07 (82.95-332.49) | 165.97 (1307.74) | 3.33 (2.35) | 120.61 (60.24) |
|  | Acute leukaemia | 10 | 8.63 (4.62-16.15) | 8.63 (66.16) | 2.20 (1.28) | 8.48 (4.54) |
|  | Lymphocytic leukaemia | 9 | 28.55 (14.54-56.04) | 28.54 (224.51) | 2.75 (1.76) | 26.85 (13.68) |
|  | Chronic myelomonocytic leukaemia | 8 | 15.29 (7.56-30.95) | 15.29 (103.21) | 2.38 (1.34) | 14.80 (7.31) |
|  | Refractory cancer | 8 | 33.67 (16.4-69.13) | 33.66 (235.40) | 2.67 (1.62) | 31.33 (15.26) |
|  | Blastic plasmacytoid dendritic cell neoplasia | 7 | 80.63 (36-180.58) | 80.6 (464.71) | 2.67 (1.49) | 68.22 (30.46) |
|  | Chloroma | 7 | 17.21 (8.09-36.64) | 17.21 (102.82) | 2.30 (1.19) | 16.59 (7.80) |
|  | Mantle cell lymphoma recurrent | 7 | 18.68 (8.77-39.81) | 18.68 (112.32) | 2.33 (1.22) | 17.95 (8.42) |
|  | Neoplasm skin | 7 | 5.25 (2.49-11.06) | 5.24 (23.77) | 1.58 (0.48) | 5.19 (2.47) |
|  | Waldenstrom'smacroglobulinaemia | 7 | 19.27 (9.04-41.09) | 19.26 (116.10) | 2.34 (1.23) | 18.49 (8.67) |
|  | B-cell small lymphocytic lymphoma | 6 | 39.79 (17.25-91.79) | 39.78 (207.92) | 2.37 (1.14) | 36.55 (15.84) |
|  | Prolymphocytic leukaemia | 6 | 187.59 (72.08-488.2) | 187.53 (779.24) | 2.52 (1.18) | 131.57 (50.55) |
|  | Abdominal neoplasm | 5 | 6.18 (2.56-14.95) | 6.18 (21.41) | 1.46 (0.16) | 6.11 (2.53) |
|  | Anal cancer | 5 | 5.08 (2.10-12.26) | 5.08 (16.18) | 1.33 (0.03) | 5.03 (2.08) |
|  | Blast cell crisis | 5 | 15.30 (6.27-37.33) | 15.30 (64.57) | 1.90 (0.59) | 14.82 (6.07) |
|  | Myeloid leukaemia | 5 | 12.58 (5.17-30.60) | 12.57 (51.78) | 1.83 (0.52) | 12.25 (5.03) |
|  | Oncologic complication | 5 | 6.53 (2.70-15.80) | 6.53 (23.08) | 1.49 (0.19) | 6.45 (2.67) |
|  | T-cell lymphoma | 5 | 6.10 (2.52-14.74) | 6.09 (21.00) | 1.45 (0.15) | 6.02 (2.49) |
|  | Cancer in remission | 4 | 20.84 (7.64-56.83) | 20.84 (72.11) | 1.74 (0.26) | 19.93 (7.31) |
|  | Mantle cell lymphoma refractory | 4 | 60.37 (21.22-171.73) | 60.35 (205.18) | 1.90 (0.37) | 53.16 (18.69) |
|  | Nasal neoplasm | 4 | 12.59 (4.66-34.03) | 12.59 (41.49) | 1.59 (0.13) | 12.27 (4.54) |
|  | Tumour associated fever | 4 | 11.75 (4.35-31.72) | 11.75 (38.30) | 1.57 (0.11) | 11.47 (4.25) |
| Respiratory, thoracic and mediastinal disorders | Pleural effusion | 145 | 2.83 (2.40-3.33) | 2.81 (169.06) | 1.46 (1.22) | 2.80 (2.38) |
|  | Lung infiltration | 27 | 5.05 (3.45-7.38) | 5.04 (86.53) | 2.08 (1.52) | 5.00 (3.42) |
|  | Atelectasis | 20 | 3.32 (2.14-5.16) | 3.32 (32.17) | 1.50 (0.85) | 3.30 (2.13) |
|  | Bronchopneumopathy | 5 | 16.58 (6.79-40.5) | 16.57 (70.50) | 1.93 (0.62) | 16.01 (6.55) |
|  | Alveolar lung disease | 4 | 17.51 (6.44-47.56) | 17.50 (59.85) | 1.69 (0.22) | 16.87 (6.21) |
| Social circumstances | Blood product transfusion dependent | 10 | 54.72 (28.35-105.62) | 54.7 (468.57) | 3.05 (2.09) | 48.73 (25.25) |
|  | Disease risk factor | 4 | 12.42 (4.60-33.54) | 12.41 (40.82) | 1.59 (0.13) | 12.10 (4.48) |
| Surgical and medical procedures | Hospice care | 491 | 57.49 (52.28-63.22) | 56.04 (23539.89) | 5.50 (5.36) | 49.79 (45.27) |
|  | Hospitalisation | 467 | 3.09 (2.81-3.38) | 3.04 (638.27) | 1.59 (1.45) | 3.02 (2.76) |
|  | Transfusion | 377 | 37.53 (33.76-41.74) | 36.81 (12122.72) | 4.96 (4.81) | 34.03 (30.61) |
|  | Platelet transfusion | 175 | 208.86 (174.39-250.15) | 206.96 (24352.55) | 6.29 (6.03) | 140.82 (117.58) |
|  | Bone marrow transplant | 105 | 116.36 (93.8-144.35) | 115.73 (9445.10) | 5.61 (5.30) | 91.73 (73.94) |
|  | Chemotherapy | 92 | 29.98 (24.26-37.05) | 29.84 (2401.18) | 4.42 (4.11) | 28 (22.66) |
|  | Stem cell transplant | 83 | 73.39 (58.14-92.63) | 73.07 (5056.31) | 5.16 (4.82) | 62.76 (49.72) |
|  | Packed red blood cell transfusion | 60 | 31.42 (24.17-40.85) | 31.33 (1644.14) | 4.3 (3.91) | 29.3 (22.54) |
|  | Transplant | 55 | 30.55 (23.23-40.17) | 30.46 (1465.43) | 4.23 (3.83) | 28.55 (21.71) |
|  | Central venous catheterisation | 25 | 8.70 (5.85-12.93) | 8.69 (166.81) | 2.67 (2.09) | 8.54 (5.75) |
|  | Infusion | 22 | 65.56 (41.88-102.64) | 65.49 (1215.19) | 3.99 (3.33) | 57.09 (36.47) |
|  | Rehabilitation therapy | 19 | 5.16 (3.28-8.12) | 5.16 (62.95) | 2.01 (1.34) | 5.11 (3.25) |
|  | Intensive care | 18 | 17.14 (10.7-27.45) | 17.12 (262.98) | 3.11 (2.41) | 16.52 (10.31) |
|  | Endotracheal intubation | 15 | 6.80 (4.08-11.32) | 6.79 (73.00) | 2.21 (1.46) | 6.71 (4.03) |
|  | Palliative care | 13 | 10.72 (6.18-18.59) | 10.71 (111.75) | 2.54 (1.73) | 10.48 (6.04) |
|  | Gastrointestinal tube insertion | 10 | 5.03 (2.69-9.38) | 5.02 (31.87) | 1.73 (0.81) | 4.98 (2.67) |
|  | Medical procedure | 10 | 7.36 (3.94-13.75) | 7.35 (54.00) | 2.07 (1.15) | 7.25 (3.88) |
|  | Fluid replacement | 9 | 37.52 (18.99-74.13) | 37.51 (294.55) | 2.84 (1.84) | 34.62 (17.53) |
|  | Allogenic stem cell transplantation | 8 | 184.32 (80.68-421.09) | 184.24 (1025.97) | 2.91 (1.75) | 129.95 (56.88) |
|  | Splenectomy | 8 | 10.74 (5.33-21.67) | 10.74 (68.96) | 2.18 (1.15) | 10.50 (5.21) |
|  | Micrographic skin surgery | 5 | 36.47 (14.65-90.83) | 36.46 (159.19) | 2.12 (0.78) | 33.74 (13.55) |
| Vascular disorders | Phlebitis | 21 | 5.67 (3.69-8.72) | 5.67 (79.66) | 2.15 (1.51) | 5.61 (3.64) |

SOC, system organ class; PT, preferred term; ROR, reporting odds ratio; CI, confidence interval; PRR, proportional reporting ratio; χ2, chi-information component; IC, information component; IC025, the lower limit of 95% CI of the IC; EBGM, empirical Bayesian geometric mean; EBGM05, the lower limit of 95% CI of EBGM.
